# Vaccination Status and the Detection of SARS-CoV-2 Infection in Health Care Personnel Under Surveillance in Long-term Residential Facilities

**DOI:** 10.1001/jamanetworkopen.2021.34229

**Published:** 2021-11-10

**Authors:** Katherine Linsenmeyer, Michael E. Charness, William J. O’Brien, Judith Strymish, Sucheta J. Doshi, Sven K. Ljaamo, Kalpana Gupta

**Affiliations:** 1 VA Boston Healthcare System, Boston, Massachusetts; 2 VA Bedford Healthcare System, Bedford, Massachusetts

## Abstract

This cohort study examines the association of vaccination status and detection of SARS-CoV-2 infection in health care personnel at long-term residential facilities.

## Introduction

Routine testing for asymptomatic SARS-COV-2 infection among health care personnel (HCP) who have not been vaccinated against COVID-19 can reduce transmission to the residents of long-term care (LTC) facilities.^1,2^ However, the utility of surveillance testing for LTC HCP who have been vaccinated against COVID-19 is unclear. Although rates of positive results among HCP who are vaccinated are extremely low,^3,4,5^ breakthrough infections and transmission still occur.^6^

The Veterans Health Administration (VHA) implemented routine surveillance of HCP and residents of LTC units in April 2020. Surveillance was intensified in January 2021, coincident with a national surge of cases and first vaccine availability. Therefore, the object of this study was to assess whether vaccination was associated with decreased detection of asymptomatic SARS-CoV-2 infection in HCP working in LTC facilities.

## Methods

This cohort study was deemed exempt from review and the requirement for informed consent by the VA Boston Healthcare System institutional review board because it was a quality improvement project and not research. The study followed the Strengthening the Reporting of Observational Studies in Epidemiology (STROBE) reporting guideline.

The study cohort included all 431 HCP working in the 105-bed LTC units at VA Boston Healthcare System and 1542 HCP in the 318-bed LTC units at the Bedford VA in Massachusetts. Mandatory surveillance testing using reverse transcription–polymerase chain reaction (RT-PCR) was conducted weekly for 1269 HCP at the Bedford LTC facility. The remaining Bedford and Boston LTC HCP underwent twice weekly antigen testing (Binax NOW; Abbott Laboratories). Positive antigen test results were confirmed by PCR. All HCP with a positive PCR result were asymptomatic or minimally symptomatic at the time of testing. We excluded LTC HCP diagnosed with SARS-CoV-2 infection outside of this surveillance system. The study period was January 15, 2021, through June 8, 2021.

We calculated descriptive statistics of the eligible testing population and incidence of positive tests, stratified by vaccination status, in 5 time periods. Analysis was performed in R statistical software version 4.0.4 (R Project for Statistical Computing). Data analysis was performed as part of ongoing quality improvement activities from January 15, 2021 through July 15, 2021.

## Results

A total of 52 557 tests were mandated among 1973 HCP. Of these, 1948 HCP had negative test results throughout the study period, and 25 HCP had positive test results (Figure). By the end of the study, 1388 of 1973 HCP (70.3%) and approximately 90% of LTC residents were fully vaccinated. Lower case rates were detected in vaccinated vs unvaccinated staff during each monthly period (Table). The overall detection rate for asymptomatic SARS-CoV-2 infection was 4 of 1388 HCP (0.3%) among those who were fully vaccinated and 21 of 585 HCP (3.6%) among those who were unvaccinated. The detection rate declined toward zero in both groups in parallel with a drop in community transmission (Figure). SARS-CoV-2 infection in 1 HCP who was not vaccinated was linked through contact tracing to asymptomatic infection in 1 resident who was fully vaccinated.

**Figure.**
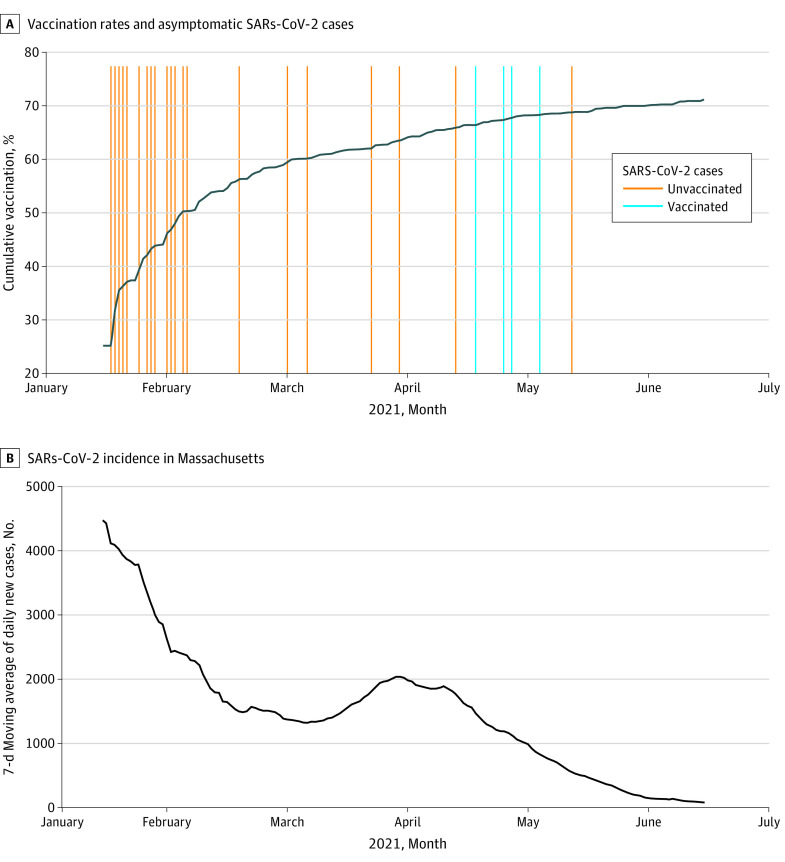
Detection of SARS-CoV-2 as a Function of Vaccination Status and Community Prevalence A, Vertical lines indicate the date of each unique positive test result for SARS-CoV-2. B, Source: Massachusetts COVID-19 response reporting.

**Table.** Monthly Rate of Positive Tests for SARS-CoV-2 Infection in Health Care Personnel Who Were Vaccinated or Unvaccinated from January to June 2021

Month	Time period	Vaccinated			Unvaccinated		
		Total tests	Positive results	Rate, %	Total tests	Positive results	Rate, %
1	January 16-February 12	5440	0	0	4472	14	0.40
2	February 13-March 13	5192	0	0	4265	3	0.07
3	March 14-April 11	6672	1	0.01	4167	1	0.04
4	April 12-May 10	7007	3	0.04	4102	2	0.02
5	May 11-June 8	7212	0	0	4028	1	0.02
Total	January 16-June 8	31 523	4	0.01	21 034	21	0.10

## Discussion

This cohort study found that frequent, mandatory surveillance of HCP in a LTC setting was effective in detecting SARS-CoV-2 infection in HCP. The yield of positive test results was much higher in HCP who were unvaccinated than those who were vaccinated, consistent with an evolving literature that suggests full vaccination status reduces asymptomatic SARS-CoV-2 infection in HCP.^3,4,5^ The observation that surveillance was primarily beneficial in HCP who were unvaccinated during periods of high community transmission is consistent with recent guidance from the Centers for Disease Control and Prevention^1^ and presents a quandary regarding how to identify and differentially test HCP who are unvaccinated in the absence of mandatory vaccination policy.

The detection of asymptomatic SARS-CoV-2 infection decreased in parallel with community prevalence. As case rates decrease, the proportion of tests with positive results that are false positives will increase, even in HCP who are unvaccinated, sometimes leading to unnecessary quarantine of essential HCP. Hence, the utility of frequent surveillance will vary as a function of the rate and trend of community transmission, vaccination status of HCP and residents, and the transmissibility of variant strains of SARS-CoV-2.^6^

A limitation of this study is the observational design involving a relatively small number of HCP from 2 facilities in a single region and a single health care system. In addition, the number of completed tests may have been less than the number of mandated tests. Frequent antigen testing should have detected most infected individuals. Sequencing data were not available; however, the predominant variant in Massachusetts and at our facilities shifted from D614G to B.1.1.7 (Alpha) between March and April 2021. Additionally, these findings may not generalize to the Delta variant, which was not studied.
